# P-2024. Multiple Cases of the Delta Substrain AY.29 with Casirivimab/Imdevimab Escape Mutation Identified in a Nosocomial COVID-19 Outbreak in a Hematology Ward

**DOI:** 10.1093/ofid/ofae631.2180

**Published:** 2025-01-29

**Authors:** Yuka Hamada, Hideki Kawamura, Naomi Shinkawa, Makoto Kuroda, Hajime Kamiya

**Affiliations:** Kagoshima Prefectural Institute for Environmental Research and Public Health, Kagoshima-city, Kagoshima, Japan; Kagoshima University, Kagoshima, Kagoshima, Japan; Kagoshima Prefectural Institute for Environmental Research and Public Health, Kagoshima-city, Kagoshima, Japan; National Institute of Infectious Diseases, Shinjuku-ku, Tokyo, Japan; National Institute of Infectious Diseases, Shinjuku-ku, Tokyo, Japan

## Abstract

**Background:**

Underlying immunodeficiencies are a concern due to the risk of persistent infections of SARS-CoV-2. The Delta Substrain AY.29 with casirivimab/imdevimab escape mutations was detected from patients who was diagnosed as a COVID-19 while hospitalized in a hematology ward. This study aims to investigate the factors leading to casirivimab/imdevimab escape mutations and to propose measures to strengthen the control of nosocomial COVID-19 infections.

**Figure 1.**
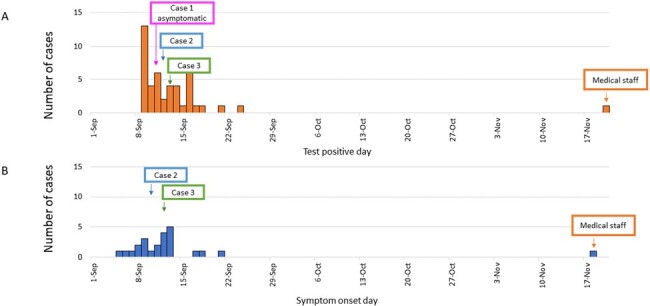
Epidemic curve based on the day of positive SARS-CoV-2 PCR test (A) and on the symptom onset day (B) of COVID-19 outbreak. Epidemic curves based on onset symptom date (B) exclude 21 cases with asymptomatic or unknown onset date. Cases 1-3 and medical staff are COVID-19 infected patients with casilivimab/imdevimab escape mutation strains detected

**Methods:**

We conducted an active epidemiological investigation to the COVID-19 outbreak occurred at the hematology ward in one regional hospital in Southern part of Japan. In addition to collecting demographic information on patients, we created a network diagram that integrates the results of genomic analyses to evaluate the factors associated with casirivimab/imdevimab escape mutation.

**Figure 2.**
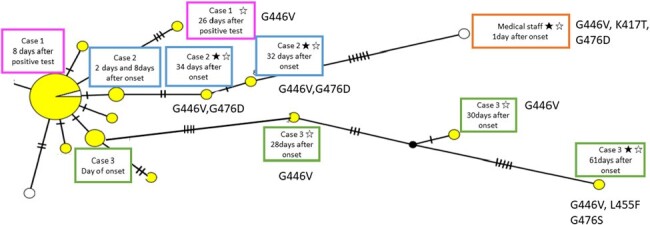
Results of genome analysis of virus isolated from cases in this COVID-19 outbreak (n=30) ★; strains with casilivimab escape mutation ⋆; strains with imdevimab escape mutation

**Results:**

In this outbreak, a total of 44 COVID-19 patients were identified (Figure 1), including 30 inpatients and 14 medical staff or family members. Initially, the strains detected from the cases were identical gene sequences of the Delta strain, and 24 cases were treated with casirivimab/imdevimab. However, three patients with lymphatic hematologic diseases 26-32 days after initial diagnosis tested positive for SARS-CoV-2 infection. The samples detected from these patients had G446V mutation, indicative of imdevimab escape mutant, with mutations indicative of casirivimab escape, such as K417T (1 strain), L455F (1 strains, G476D (2 strains), and G476S (1 strain) (Figure 2). The medical staff, who had been working in the dialysis unit prior to the outbreak, first became symptomatic two months after the outbreak and was diagnosed with COVID-19 by PCR. His samples revealed a mutant strain detected in three patients.

**Conclusion:**

The results suggest that patients with immunocompromised, especially lymphatic diseases, who being treated with casirivimab/imdevimab may not be sufficiently eradicated the virus from the body even if their symptoms improve. This may have caused the virus acquire mutations. When casirivimab/imdevimab are administered to immunocompromised patients, it is important that periodic viral testing should be performed. Accurate infection control measures must be implemented to prevent the spread of the mutant strain unnoticed.

**Disclosures:**

All Authors: No reported disclosures

